# Direct standardization method according to Robson classification for comparison of cesarean rates

**DOI:** 10.1186/s12884-023-05416-9

**Published:** 2023-02-16

**Authors:** Marcelle Gonçalves Campos, Ana Beatriz Franco-Sena, Fernanda Rebelo

**Affiliations:** 1 Postgraduate Program in Children’s and Women’s Health, Oswaldo Cruz Foundation, National Institute of Women’s, Children’s and Adolescents’ Health Fernandes Figueira, Rio de Janeiro, RJ Brazil; 2 Faculty of Nutrition Emília de Jesus Ferreiro, Department of Social Nutrition, Fluminense Federal University, Niterói, RJ Brazil; 3 Clinical Research Unit, Oswaldo Cruz Foundation, National Institute of Women’s, Children and Adolescents’ Health Fernandes Figueira, Rio de Janeiro, RJ Brazil

**Keywords:** Cesarean section, Robson ten-Group classification, Direct standardization, Epidemiology

## Abstract

**Background:**

Compare cesarean section rates between populations or within a population over time using the crude measure is biased mainly due to differences in the characteristics of the obstetric population. The Robson Ten Group Classification (RTGC) is being widely used all over the world based on a few basic obstetrics variables.

**Objectives:**

Propose a method of direct standardization according to RTGC to make the overall rates of cesarean sections comparable between different populations or within the same population over time.

**Methods:**

We used data from the WHO Global Maternal and Perinatal Health Survey (WHOGS) conducted between 2004 and 2008 and data from the WHO Multinational Survey on Maternal and Neonatal Health (WHOMCS) conducted between 2010 and 2011, covering information from obstetric population of 21 countries. The standard population was based in the average size of Robson Groups in WHOMCS. The crude and standardized rates, their differences intra and inter populations, and its respective confidence intervals were calculated.

**Results:**

The impact and importance of the method were demonstrated. The five leading countries list on cesarean rates was completely modified and changes of cesarean rates over time in the same country varied in both directions by the standardization.

**Conclusion:**

This method is useful to compare overall rates as an additional information when RTGC Report Table is been used or, for some type of studies as analytical ecologic studies with multiple groups, where leading with the report tables are laborious and hard to interpret. The use of Robson Ten Group Classification for direct standardization of cesarean rates is easy to apply and interpret.

**Supplementary Information:**

The online version contains supplementary material available at 10.1186/s12884-023-05416-9.

## Background

Global cesarean section (CS) rate is approximately 21.1%, but there are significant differences in the access of CS all over the world. In least developed countries, CS represent about 8.2% of births, including sub-Saharan Africa with the lowest rate (5.0%). Meanwhile, the five countries with highest overall CS rates worldwide are Dominican Republic (58.1%), Brazil (55.7%), Cyprus (55.3%), Egypt (51.8%) and Turkey (50.8%), where the surgical deliveries surpass vaginal births [1].

It is agreed that the comparison of CS rates between populations or in the same population over time is problematic. The different characteristics of the obstetric population must be taken into account to avoid bias. To assist with this issue, many classifications for CS have been proposed over the years. In a systematic review, Torloni et al. [2] suggested the Robson Ten-Group Classification (RTGC) as the best option to meet local and international needs.

The RTGC is based on five parameters (obstetric history, onset of labor, fetal presentation, number of neonates and gestational age) to define ten mutually exclusive and fully inclusive groups (Table 1). The classification is based on a few routinely recorded variables and can be applied prospectively [3].

**Table 1:** Parameters for Robson Ten-Group Classification

Group	Obstetric history	Onset of labor	Fetal presentation	Number of neonates	Gestational age (weeks)
**1**	Nulliparous	Spontaneous	Cephalic	Single	≥37
**2**	Nulliparous	Induced or caesarean section before labor	Cephalic	Single	≥37
**3**	Multiparous without a previous uterine scar	Spontaneous	Cephalic	Single	≥37
**4**	Multiparous without a previous uterine scar	Induced or caesarean section before labor	Cephalic	Single	≥37
**5**	Previous cesarean section	–	Cephalic	Single	≥37
**6**	Nulliparous	–	Breech	Single	–
**7**	Multiparous	–	Breech	Single	–
**8**	–	–	–	Multiple	–
**9**	–	–	Transverse or oblique lie	Single	–
**10**	–	–	Cephalic	Single	< 37

In 2015, the World Health Organization (WHO) published an Implementation Manual for RTGC use to assess, monitor and compare CS rates [3]. The manual proposes the construction of the Robson Classification Report Table for each population / time lapse and serial steps to interpret it, including data quality, type of population and assessment of CS rates. Therefore, it is a holistic and irreplaceable approach to deal with the complexity of the factors that influence CS rates. However, in certain situations in which it is necessary to work with the overall rate, the presentation and use of Report Table may not be applicable.

In recent years, many papers have been published comparing the overall CS rates as part of the results, even when data for RTGC is available [4–9]. However, CS rates are expected to be different between RTGC groups, which makes meaningless and biased comparison of overall crude rates among populations with distinct RTGC distributions.

Understanding the complexity of factors that influence CS rates, the aim of this study is to propose a direct standardization method according to Robson’s classification in order to make overall CS rates comparable among different populations or in the same population over time. Researchers will benefit from having a single measure to depict CS rates. It will be useful when comparing overall rates in addition to the Robson Classification Report Table, or for some type of studies such as multi-group analytical ecological studies that deal with the Report Tables which are laborious and hard to interpret.

## Methods

### Direct standardization

Direct standardization was originally developed to adjust age when comparing mortality and morbidity rates [10]. Although it is not a novel method and some authors have already implemented it to deal with CS rates [11–13], it has never been used based on RTGC.

In order to apply this method it is necessary to define a standard population. This population must be stratified by RTGC groups and will work as a weighting factor. In addition, the RTGC-specific CS rates for each of the populations to be compared will be applied in the standard population. The sum of these results for each population is the total number of CS that would have been expected if the populations had had identical distributions according to RTGC. By dividing this expected number of CS by the total standard population at the various populations will yield a standardized or RTGC-adjusted CS rate.

The directly RTGC-adjusted CS rate (ACS) for populations X and Y can be mathematically represented by the following equations:

$${ACS}_x=\sum \left({r}_{ix}{n}_{is}\right)/\sum {n}_{is}$$

$${ACS}_y=\sum \left({r}_{iy}{n}_{is}\right)/\sum {n}_{is}$$

The population is *n*_*is*_ in the *i*th Robson group of the standard population, *r*_*ix*_ and *r*_*iy*_ are the CS rates in Robson group *i* in populations X and Y, respectively.

### The standard population

The population distribution according to RTGC standard will influence the comparison between populations, as exemplified in the WHO analysis of age-adjusted mortality rates [14]. The choice is arbitrary and there is no conceptual justification or theoretical formula to define standards populations. However, following the WHO strategy for age standardization, it would be preferable to find a standard that reflects the average structure of all populations [14].

For the present study, we are using a standard population based on data publicly available in WHO Multi-Country Survey of Maternal and Newborn Health (WHOMCS; 2010–11) [15]. This cross-sectional research aimed to characterize severe maternal, perinatal and neonatal morbidity, with emphasis on maternal near-miss indicators. It was done between May 2010 and December 2011 and involved a worldwide network of health facilities, in 29 countries. Data from 21 of those countries were published by Vogel et al. [9], specifying their relative size of obstetric population according to RTGC. As data were presented stratified by Human Development Index (HDI), the weighted mean was calculated to achieve the relative size of Robson Groups for the complete sample and is presented in Table 2. The relative size of Robson Groups was applied to an arbitrary population of 100,000 people, determining the absolute size of each Robson Group in the standard population. The establishment of an international standard population is desirable, but it is not the aim of this study and needs to be discussed in detail by a team of experts.

**Table 2:** Relative size of Robson groups for 21 countries participants in WHO Multi-Country Survey of Maternal and Newborn Health (WHOMCS; 2010 – 2011) and standard population based on its average

Robson Group	Relative size of Robson groups according to HDI (%)			Weighted mean^**a**^	Standard Population^**b**^
	High	Moderate	Low		
1	24.00	34.40	29.10	29.41	29,406
2	13.50	8.10	3.90	8.70	8703
3	25.00	30.10	43.70	32.29	32,288
4	8.20	3.70	3.10	5.03	5033
5	12.40	8.90	7.30	9.61	9612
6	1.60	1.90	0.90	1.51	1513
7	1.50	1.80	1.70	1.67	1671
8	1.30	1.40	1.90	1.51	1510
9	1.70	0.70	0.70	1.03	1034
10	6.60	7.10	6.40	6.73	6732
x	4.20	1.90	1.30	2.50	2497
Total	100	100	100	100	100,000

Data from WHO Multi-Country Survey of Maternal and Newborn Health (WHOMCS), processed by Vogel et al. [9]

*HDI* Human Development Index, *x* unable to classify into Robson groups

^a^Weighted mean considered that high, moderate and low HDI countries accounted for 33.4, 37.9 and 28.6% of the total population, respectively

^b^Standard population was determined based on the relative size of Robson groups for all countries (weighted mean), considering a total population of 100,000 units

### Application

The method was applied to standardize the data presented in the paper of Vogel et al. [9], which by its turn, used the data from WHO Global Survey of Maternal and Perinatal Health (WHOGS; data collected between 2004 and 2008) and the WHOMCS. Twenty-one participating countries were identified in both surveys [15, 16].

Crude CS rates were calculated as the total number of CS divided by the number of deliveries for each country. Standardized CS rates were calculated as the total number of standardized CS divided by the total deliveries of standard population (100,000). A ranking was created to compare the crude rates with the standardized rates and identify the variation in the position of the countries with the standardized rates.

The 95% confidence interval of the raw and standardized rates was calculated according to the formula:

$$\overline{X}\pm Z\ast \frac{\sigma }{\surd n}$$

Where X̅ is the sample mean, Z is the value for a 95% confidence level, σ is the population standard deviation and *n* is the sample size. The impact of standardization was considered statistically significant if the 95% confidence intervals did not overlap.

To observe the variation within populations over time, the difference between WHOGS and WHOMCS CS rates and its respective 95% confidence intervals were calculated for each country, both for crude and adjusted measures. The variation of CS rates within countries was considered to be significantly affected by standardization when the confidence intervals of crude and standardized differences did not overlap.

## Results

**Fig. 1:**
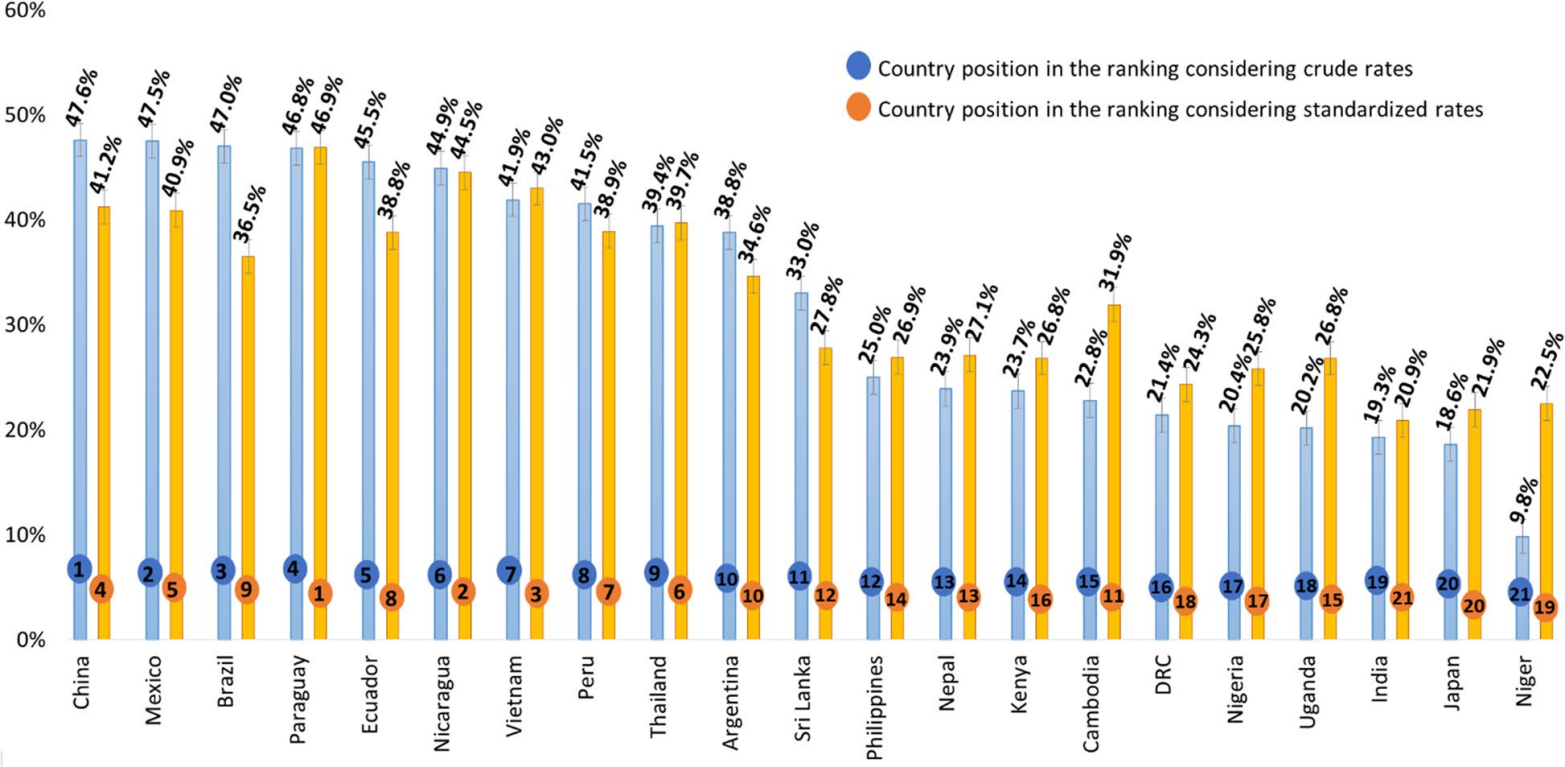
Directly RTGC-standardized cesarean rates for countries participants in WHOMCS and ranking of crude and standardized rates. Data from WHO Multi-Country Survey of Maternal and Newborn Health (WHOMCS), processed by Vogel et al. [9]. The blue color indicates the crude rate value and the orange color indicates the standardized rate value

Figure 1 presents the CS rate for each country, crude and standardized by the RTGC. In addition, countries were ranked from highest to lowest CS according to the crude and standardized estimate. Comparing crude CS rates, the leading country is China with 47.6%, followed by Mexico (47.5%), Brazil (47.0%), Paraguay (46.8%) and Ecuador (45.5%). On the other hand, comparing standardized rates, the leading country is Paraguay with 46.9%, followed by Nicaragua (44.5%), Vietnam (43.0%), China (41.2%) and Mexico (40.9%). Brazil presented the biggest difference regarding its position in CS rank, from third place in the crude rate to ninth place in the standardized rate.

Table 3 shows the difference, for the crude and RTGC standardized rates, between the WHOMCS and the WHOGS data, in order to observe the implication of the standardization of CS rates in the same population over time. All countries, except for Nicaragua, Paraguay and Thailand, had their WHOMCS rates influenced by standardization. All countries, except for Peru, Thailand and Vietnam, had their WHOGS rates impacted by standardization (Table 3). Brazil, DRC, Ecuador, Kenya, Mexico, Nicaragua, Niger, Peru, Philippines and Uganda had their rates difference (WHOMCS rate – WHOGS rate) significantly affected by standardization.

**Table 3:** Crude and directly RTGC-standardized cesarean rates for 21 countries participants of the WHO Global Survey of Maternal and Perinatal Health(WHOGS, 2004 - 2008) and WHO Multi-Country Survey of Maternal and Newborn Health (WHOMCS, 2010 – 2011)

	WHOGS (%)		WHOMCS (%)		Difference^**a**^ (%)	
	Crude (IC 95%)	Standardized (IC 95%)	Crude (IC 95%)	Standardized (IC 95%)	Crude (IC 95%)	Standardized (IC 95%)
**Argentina**^d,c^	**35.1 (34.2, 36.0)**	**32.0 (31.7, 32.3)**	**38.8 (37.9, 39.8)**	**34.6 (34.3, 34.9)**	3.7 (2.4, 5.0)	2.6 (2.2, 3.0)
**Brazil**^b,d,c^	**27.0 (25.8, 28.2)**	**24.0 (23.8, 24.3)**	**47.0 (45.7, 48.3)**	**36.5 (36.3, 36.8)**	**20.0 (18.3, 21.7)**	**12.5 (12.1, 12.9)**
**Cambodia**^d,c^	**14.7 (13.8, 15.6)**	**22.7 (22.5, 23.0)**	**22.8 (21.6, 24.0)**	**31.9 (31.6, 32.2)**	8.1 (6.6, 9.6)	9.2 (8.8, 9.5)
**China**^d,c^	**46.2 (45.4, 47.0)**	**40.9 (40.7, 41.3)**	**47.6 (46.7, 48.4)**	**41.2 (40.9, 41.5)**	1.4 (0.2, 2.6)	0.2 (−0.3, 0.6)
**DRC**^b,d,c^	**13.1 (12.4, 13.8)**	**17.6 (17.3, 17.8)**	**21.4 (20.5, 22.3)**	**24.3 (24.0, 24.5)**	**8.2 (7.0, 9.4)**	**6.7 (6.4, 7.1)**
**Ecuador**^b,d,c^	**40.3 (39.5, 41.2)**	**38.5 (38.2, 38.8)**	**45.5 (44.5, 46.5)**	**38.8 (38.5, 39.1)**	**5.2 (3.9, 6.5)**	**0.4 (−0.1, 0.8)**
**India**^d,c^	**17.7 (17.3, 18.2)**	**20.2 (19.9, 20.4)**	**19.3 (18.9, 19.8)**	**20.9 (20.6, 21.1)**	1.6 (1.0, 2.3)	0.7 (0.4, 1.1)
**Japan**^d,c^	**19.8 (18.5, 21.2)**	**22.0 (21.7, 22.2)**	**18.6 (17.3, 19.9)**	**21.9 (21.7, 22.2)**	−1.2 (−3.1, 0.6)	−0.0(−0.4, 0.4)
**Kenya**^b,d,c^	**16.0 (15.4, 16.5)**	**22.3 (22.0, 22.5)**	**23.7 (23.1, 24.3)**	**26.8 (26.6, 27.1)**	**7.7 (6.7, 8.5)**	**4.6 (4.2, 5.0)**
**Mexico**^b,d,c^	**39.8 (39.0, 40.6)**	**36.4 (36.1, 36.7)**	**47.5 (46.6, 48.4)**	**40.9 (40.6, 41.2)**	**7.7 (6.5, 8.9)**	**4.5 (4.1, 4.9)**
**Nepal**^d,c^	**20.4 (19.5, 21.3)**	**25.0 (24.7, 25.3)**	**23.9 (23.1, 24.7)**	**27.2 (26.9, 27.4)**	3.5 (2.3, 4.6)	2.2 (1.8, 2.5)
**Nicaragua**^b,d^	**26.7 (25.5, 28.1)**	**28.9 (28.6, 29.2)**	44.9 (43.5, 46.2)	44.5 (44.2, 44.8)	**18.1 (16.2,20.0)**	**15.6 (15.2, 16.0)**
**Niger**^b,d,c^	**5.3 (4.9, 5.8)**	**11.5 (11.3, 11.7)**	**9.8 (9.3, 10.4)**	**22.5 (22.3, 22.8)**	**4.5 (3.7, 5.8)**	**11.0 (10.7, 11.4)**
**Nigeria**^d,c^	**14.5 (13.7, 15.2)**	**19.8 (19.5, 20.0)**	**20.4 (19.7, 21.2)**	**25.8 (25.5, 26.0)**	6.0 (4.9, 7.0)	6.0 (5.6, 6.3)
**Paraguay**^d^	**41.9 (40.2, 43.5)**	**39.6 (39.3, 39.9)**	46.8 (45.2, 48.5)	46.9 (46.6, 47.2)	5.0 (2.7, 7.3)	7.3 (6.9, 7.8)
**Peru**^b,c^	34.3 (33.6, 35.1)	34.2 (33.9, 34.5)	**41.5 (40.7, 42.2)**	**38.9 (38.6, 39.2)**	**7.1 (6.1, 8.2)**	**4.6 (4.2, 5.1)**
**Philippines**^b,d,c^	**17.9 (17.2, 18.7)**	**23.7 (23.5, 24.0)**	**25.0 (24.2, 25.8)**	**26.9 (26.6, 27.2)**	**7.0 (5.9, 8.1)**	**3.1 (2.8, 3.5)**
**Sri Lanka**^d,c^	**29.9 (29.1, 30.6)**	**23.8 (23.6, 24.1)**	**33.0 (32.3, 33.7)**	**27.8 (27.5, 28.1)**	3.1 (2.1, 4.1)	4.0 (3.6, 4.3)
**Thailand**	34.1 (33.1, 35.0)	34.6 (34.3, 34.9)	39.4 (38.4, 40.5)	39.7 (39.4, 40.0)	5.4 (4.0, 6.8)	5.0 (4.6, 5.4)
**Uganda**^b,d,c^	**15.1 (14.4, 15.7)**	**23.5 (23.2, 23.7)**	**20.2 (19.4, 21.0)**	**26.9 (26.6, 27.1)**	**5.1 (4.1, 6.2)**	**3.4 (3.0, 3.8)**
**Vietnam**^c^	35.9 (35.1, 36.7)	36.6 (36.3, 36.9)	**41.9 (41.1, 42.7)**	**43.0 (42.7, 43.3)**	6.1 (4.9, 7.2)	6.4 (5.9, 6.8)

Data from WHO Global Survey of Maternal and Perinatal Health (WHOGS) and WHO Multi-Country Survey of Maternal and Newborn Health (WHOMCS), processed by Vogel et al. [9]

^**a**^Difference = (WHOMCS rate – WHOGS rate)

^b^Variation between WHOMCS and WHOGS rates were significantly impacted by standardization

^c^WHOMCS rates were significantly impacted by standardization

^d^WHOGS rates were significantly impacted by standardization. Values marked in bold highlight the significant results

When comparing crude rates, the largest increase in CS over time was observed in Brazil (20.0%) followed by Nicaragua (18.1%). Both countries also presented the greatest increases when assessing the standardized differences (12.5 and 15.6%, respectively). However, the magnitude of the observed increase was significantly lower.

## Discussion

With the standardization, the classification of countries according to CS rates changed considerably. As example, we observed that Brazil and Niger were the countries that presented the greatest difference between crude and adjusted rates, changing from 3rd to 9th place and from 21st to 19th place in the ranking, respectively. In the same way, variations of CS rates over time in the same country were also modified by standardization. As example, the CS rates in Brazil raised 20.0% when considering crude values and only 12.5% when considering the standardized rates.

According to Betrán et al. [17], the increase in the rate of CS without true medical need does not confer a gain on health, but it causes adverse results. In addition, it may increase the demand for CS in future pregnancies that could be avoided. Identifying this profile is important to build efforts to reduce this index. Confirming this point of view, the WHO concludes that efforts should focus on ensuring that CS are performed in cases that are necessary. Only when indicated for medical reasons, and not just aiming to achieve a specific rate [3]. In this context, studies that assess factors associated with CS rates or the impact of CS rates on adverse outcomes are important tools to control and manage of this problem in public health. Fear of pain, the idea that CS are safer for the baby, comfort for health professionals and the mother, and fear of medical litigation are associated with an increase in the CS rate due to non-medical factors. In addition, changes in the obstetric population such as prevalence of obesity, maternal age, health conditions presented during pregnancy (gestational diabetes and HIV infection, number of prenatal consultations and fetal presentation) are characteristics that influence CS rates [17–19]. Therefore, standardization is necessary so that heterogeneous populations become comparable and enabling the real identification of changes in the prevalence of CS in a global context [20].

In the past, some authors have already proposed and tested methodologies to standardize CS rates. Lieberman et al. [21] described the use of direct standardization to compare CS rates between community-based and hospital-based practice settings in a teaching hospital. The percentages of women in each subgroup were determined according to obstetric history and conditions such as number of neonates, fetal presentation, gestational age and medical indication, which had many similarities with the parameters of RTGC. Hanley et al. [22] used logistic regression modeling to standardize CS rates by maternal characteristics (age, body mass index, gestational weight gain, smoking and parity) and conditions (hypertensive disorders in pregnancy, preexisting or gestational diabetes, gestational age, prior fetal or neonatal death, fetal presentation and number of neonates). Bailit and Garret [11] compared risk-adjusted methodologies for CS rates and found substantial agreement in the rankings from direct standardization and logistic regression methods. The authors reinforce that direct standardization is an easier method to comprehend and perform, what makes it a better decision on the condition that there are births in all risk strata.

With that being said, in 2015 the WHO released its statement on CS rates and proposed that the RTGC should be used as a standard instrument worldwide in the assessment, monitoring and comparison of CS rates over time. However, until the present study there is no reference on the RTGC use as a stratifying factor for standardization [23]. Standardization by RTGC merges the practice of standardization, already shown to be essential in comparing CS rates, with the standard instrument adopted by the WHO, taking an important step towards the unification of methods for CS rates comparison.

In the present study, considering only WHOMCS results, Niger and Brazil were the countries that had the greatest variation between crude and standardized rates. With the standardization, Brazil had a 10.5% drop and Niger showed a 12.7% rise in CS rates. This means that the proportion of CS in Brazil is originally high and in Niger is low, but the characteristics of its obstetric population partially justifies these numbers, and must be considered when compared with CS rates of other countries. Moreover, the relative size of Robson groups in these countries are very different from the relative sizes of the standard population, which explains the great variance between crude and standardized rates. In that regard, it is also important to note that the adjusted rate will vary according to the defined standard population [10].

For example, more than 60% of Niger obstetric population is in Robson group 3 (Multiparous [excluding previous CS], single, cephalic, > = 37 weeks, in spontaneous labour) and only 4.8% of this group have CS. For other groups, like 2, 4 and 5, that all together represent only 5.3% of the obstetric population, CS rates reach more than 50% [9]. Contrarily, group 3 of the standard population represents 32.3% of the total, while groups 2, 4 and 5 together are 23.3% of the total. In Paraguay, the country that presented the smallest variation between crude and adjusted rates, more than 30% of the obstetric population in Paraguay is in the Robson group 1 (nulliparous, with a single fetus, cephalic, > = 37 weeks, in labor spontaneous) and 36% of this group has CS. In other groups, such as 8 and 9, which together represent 1.6% of the obstetric population, the CS rates are 100%. However, standardized population group 1 contributes 29.4% to the overall CS rate and groups 8 and 9 together contribute 2.5% to the overall CS rate. In that country, the relative size of Robson’s groups is very close to the relative size of the standardized population, which justifies their smaller variation between rates.

Among the strengths of this study, we can highlight the large sample size studied in several countries and the possibility of comparison over time. As limitations, we cannot control the inconsistency of missing values and correct classification in the Robson groups in the different countries in the sample. The data used for the study is from 2011, but we do not consider it as a limitation, as the objective of the study was not to evaluate CS rates or to bring new data, but to test the methodology of direct standardization.

Finally, it is important to highlight that the standardization is only recommended for the purpose of comparisons. Planning health actions based on standardized CS rates, eliminating specificities such as territorial reality, culture and social conditions would lead to a biased analysis that could negatively affect health services [24–27]. For issues such as management and planning of health services for specific location and time, standardization is not indicated and considering the real crude numbers is mandatory [24].

## Conclusions

Our results highlight the need of standardization for comparison of CS rates. Analysis comparing crude rates of CS are biased, as they ignore the characteristics of each obstetrical population and the demand of necessary CS according to these characteristics.

Although many authors have raised the problem of comparing crude rates of CS [20–22], the adjusting methods proposed included complex modeling, were dependent of variables not routinely available, being mainly applicable for comparison between hospital units. The RTGC has been widely used all over the world, it is based on a few basic obstetrics variables and its use for a direct standardization is not only easy to apply and interpret, but also is attuned with WHO recommendation in 2015, and may complement the results of studies using the Robson Classification Report Table.

## Supplementary Information

**Additional file 1.** Data and materials. Data used to generate the results.

## Data Availability

All data generated or analyzed during this study are included in this published article and its supplementary information files (see Additional file 1).

## Abbreviations

CS: Cesarean section
RTGC: Robson Ten Group Classification
WHOGS: WHO Global Maternal and Perinatal Health Survey
WHOMCS: WHO Multinational Survey on Maternal and Neonatal Health
HDI: Human Development Index
